# Understanding the Aroma Profiles of Hui Li Red Sichuan Pepper (*Zanthoxylum bungeanum* Maxim) Across Harvesting Periods Using Sensory Evaluation, E-Nose and GC-IMS Techniques

**DOI:** 10.3390/foods14132285

**Published:** 2025-06-27

**Authors:** Lian He, Sook Wah Chan, Sze Ying Leong, Mingyi Guo, Zhiyong Hou, Xiangbo Xu, Nallammai Singaram, Dan Lin, Xing Qiao, Lin Wang, Huachang Wu, Zongyuan Lu

**Affiliations:** 1 Cuisine Science Key Laboratory of Sichuan Province, College of Culinary and Food Science Engineering, Sichuan Tourism University, 459 Hongling Road, Longquanyi District, Chengdu 610100, China; 2 School of Biosciences, Faculty of Health and Medical Sciences, Taylor’s University, Subang Jaya 47500, Selangor, Malaysia; sookwah.chan@taylors.edu.my (S.W.C.);; 3 Food Security & Nutrition Impact Lab, Taylor’s University, Subang Jaya 47500, Selangor, Malaysia; 4 Clean Technology Impact Lab, Taylor’s University, Subang Jaya 47500, Selangor, Malaysia; 5 Herbal Health Research Institute, Sichuan Tourism University, 459 Hongling Road, Longquanyi District, Chengdu 610100, China

**Keywords:** Sichuan pepper, sensory analysis, electronic nose, gas chromatography-ion mobility spectrometry, multivariate statistical analysis, PLS-DA, 24 solar terms

## Abstract

This study investigated aroma changes in Hui Li red Sichuan pepper across five different harvesting times within their typical optimum period based on 24 traditional solar terms, employing sensory evaluation, electronic nose (E-nose), gas chromatography-ion mobility spectrometry (GC-IMS) combined with relative odour activity value (ROAV) and partial least squares discriminant analysis (PLS-DA). Sensory analysis indicated that peppers were characterised by green, citrus, minty, sweet, woody, and peppery numbing aroma attributes. E-nose revealed the greatest aroma difference in peppers occurred between the early and late optimum harvest stages. GC-IMS identified 71 volatile compounds, with esters being the most abundant. Six key compounds identified were crucial for distinguishing peppers harvested at different times. Findings provided a valuable contribution to decide the optimal harvest window for Hui Li red Sichuan peppers, maximising their applications in the seasoning industry.

## 1. Introduction

Sichuan pepper belongs to the genus Zanthoxylum within the Rutaceae family. It is an aromatic plant native to China and has been used for centuries in Chinese cuisine for its distinctive aroma and the tingling, numbing sensation it imparts to dishes [1]. Sichuan pepper is used for its ability to remove fishy odours, enhance aroma and taste, and stimulate appetite [2]. Additionally, it has been found to inhibit the formation of heterocyclic amines in grilled meats, thereby contributing to food safety [3]. Currently, the planting area exceeds 1.67 million hectares in China. Over 60 varieties of Sichuan peppers have been cultivated in China, with their usage history spanning over 2600 years [4]. Notable varieties of Sichuan pepper in China include Hanyuan, Sichuan Southern, Gansu Fujiao, and Hancheng Dahongpao [5,6]. Each variety is characterised by its own unique aroma profile and attributes, influenced by growing conditions, soil, and climate. Hui Li Sichuan pepper, for example, is known for its intense aroma and numbing sensation, making it highly prized in Sichuan cuisine [7].

Sichuan pepper (*Zanthoxylum bungeanum* Maxim) contains a rich and diverse array of chemically active compounds that are closely associated with its characteristic aroma and numbing sensation. These include amide compounds, volatile oils, flavonoids, amino acids, alkaloids, and lignans [8,9,10]. The volatile oils contribute to the aroma, while the alkaloids and amides, especially hydroxy-alpha sanshool, are responsible for the unique numbing or tingling sensation that sets Sichuan pepper apart from other spices [11]. Based on the colour of their pericarp, the pepper is classified as either red Sichuan pepper (*Z. bungeanum*), commonly known as Sichuan pepper, or green Sichuan pepper (*Zanthoxylum schinifolium* Siebold & Zucc.). The distinct aroma between green and red Sichuan pepper varieties are attributed to differences in the composition and concentration of terpenes in their volatile oils, likely due to variations in the expression patterns of terpene synthesis genes [12]. The work of Feng et al. [13] reported that the higher content of linalyl acetate and limonene in the red pepper variety imparts a pronounced floral, citrusy, and lemony aroma, while the elevated levels of linalool and sabinene in the green pepper variety impart a more herbal, pine-like, and fresh aroma.

The global demand for regional spices like Sichuan pepper is rising, driven by culinary diversification and health-conscious trends. Aroma is a crucial organoleptic factor in assessing the quality of Sichuan peppers, with factors such as variety, cultivation region, harvest timing, and post-harvest processing methods all influencing their aroma profile [14,15,16]. It has been shown the fatty acid content of Sichuan pepper seeds can shape their unique aroma; with green Sichuan pepper containing high levels of palmitic acid and red Sichuan pepper rich in oleic acid, thus differentiating the aroma profiles of these two varieties [17]. Environmental factors during cultivation of Sichuan peppers, such as latitude, temperature, sunlight exposure, soil composition, and rainfall, further impact their fatty acid compositions, thereby affecting their aroma profiles [17]. Additionally, post-harvest processing of Sichuan peppers, particularly drying methods, can play a significant role in their aroma retention. For example, microwave drying of Sichuan peppers at 450 W has been found suitable for preserving their volatile compounds and, thus, their aroma characteristics, due to the short processing time [18]. Moreover, heating Sichuan peppers at 130 °C for 20 min enhances linalool levels, resulting in a stronger floral and citrus aroma [19].

The developmental stages of Sichuan pepper can be broadly divided into six stages: bud emergence, flowering, fruiting, bulking, oil gland development, and harvest [1]. The harvesting of Sichuan pepper typically takes place from July to September in China, with the harvest timing being crucial for ensuring both yield and quality. Harvesting them too early can result in underdeveloped seeds and lower yields, while harvesting too late leads to a high amount of cracked fruits, ultimately resulting in Sichuan peppers of inferior quality [20]. Currently, farmers primarily rely on their own visual and smell assessments to determine the appropriate harvesting time for Sichuan peppers, which can be challenging, especially when dealing with peppers exhibiting similar aromatic profiles. Such subjective method may not always capture the subtle differences in Sichuan peppers, leading to poor decision-making, missing the optimal harvest time for premium quality peppers, and ultimately resulting in the harvest of low-quality peppers. Therefore, establishing a scientific approach to harvest Sichuan pepper by integrating sensory evaluation with data from scientific instruments has not yet been attempted and is currently lacking, but would be valuable for improving accuracy and consistency in determining the optimal harvesting time for Sichuan peppers.

The 24 solar terms are a traditional Chinese method of calculating time, dividing the solar year into 24 equal periods based on the position of the sun in the astrological calendar (Supplementary Material Figure S1). Each solar term is associated with specific weather patterns and agricultural activities, helping farmers determine the optimal times for planting, harvesting, and other agricultural tasks [21]. Building on this premise, the present study employed the 24 traditional Chinese solar terms as a framework to guide the harvesting seasons, with the objective of investigating the aroma profiles and volatile composition in Hui Li red Sichuan peppers harvested at five selected time points within these solar terms. Firstly, a formal sensory evaluation with trained panellists was conducted to assess the aroma, taste, flavour and chemesthetic sensation of Sichuan peppers harvested at different time points, followed by the use of an electronic nose (E-nose) to distinguish the overall aroma profile of the peppers. Subsequently, both qualitative and quantitative analyses of the volatile compounds in these peppers were conducted using gas chromatography-ion mobility spectrometry (GC-IMS). The relative odour activity value (ROAV) method was employed to quantify the contribution of individual volatile compounds to the overall aroma of Sichuan peppers at each selected harvest period. Correlation analysis was then conducted to establish associations between the sensory evaluation results with trained panellists and the volatile compounds with ROAV > 1, thereby identifying key volatiles that significantly impact sensory perception. Specifically, the novelty of this study lies in its use of multivariate statistical analysis, involving unsupervised approach with principal component analysis (PCA) to examine natural clustering in the data collected from the rapid E-nose analysis, as well as supervised approach using partial least squares discriminant analysis (PLS-DA) to maximise separation of volatile compounds based on GC-IMS analysis. Then, the calculation of Variable Importance in Projection (VIP) scores allows the identification of key volatile compounds that distinguish the aroma profiles of Hui Li red Sichuan peppers across different harvest periods, offering insights into how harvest timing influences their aroma.

## 2. Materials and Methods

### 2.1. Harvesting Hui Li Red Sichuan Peppers

Whole Hui Li red Sichuan peppers, cultivated in the Liangshan Yi Autonomous Prefecture, Sichuan Province, China (latitude 102° E, longitude 26° N, 1900–2100 m altitude), were harvested for this study. The harvest periods were based on the 24 traditional Chinese solar terms (Supplementary Material Figure S1), specifically focusing on Greater Heat, Beginning of Autumn, End of Heat, White Dew, and Autumn Equinox. These terms served as key temporal markers for the five independent harvest time points of the Sichuan pepper sample. The corresponding Gregorian calendar dates for the harvest were 24 July, 7 and 22 August, and 6 and 21 September of the year 2022. The Sichuan pepper samples were sequentially numbered according to their harvest period, as follows: LSA, LSB, LSC, LSD, and LSE (Table 1). For each harvest time, approximately 1 kg of freshly harvested whole Hui Li red Sichuan pepper samples was collected from three randomly selected branches of individual trees spaced ≥10 m apart within the same orchard (Taiping Village, Taiping Town, Huili City, Liangshan Prefecture, Sichuan Province). The peppers were then pooled and dehydrated in ventilated and shaded areas for at least 24 h. After that, the samples were vacuum-sealed and transported to the food laboratory at Sichuan Tourism University for storage in cool, dark conditions until analysis.

### 2.2. Sensory Evaluation of Hui Li Red Sichuan Peppers with a Trained Sensory Panel

The sensory evaluation was approved by the Human Ethics Committee of Taylor’s University (Reference No: HEC 2024/307). Comprehensive information about the study’s requirements and potential risks was fully disclosed to all participants. Their written consent was obtained before the commencement of the study. We pledged not to release any participant’s data without their prior knowledge and consent. Furthermore, participants were given the liberty to withdraw from the study at any point they wished. Ten experienced panellists (five women and five men, n = 10, aged 18–25 years) with over one year of sensory evaluation experience were recruited. All panellists were in good health, non-smokers, had no history of rhinitis, and frequently consumed cuisine seasoned with Sichuan pepper. Prior to the formal sensory evaluation of samples, the panel received a total of 24 h of Sichuan pepper specific training (16 sessions × 1.5 h per session), based on the work of Yang et al. [20] with modifications, which included vocabulary development to define and discuss the sensory attributes of Hui Li red Sichuan peppers, comprising aroma, taste, flavour, and chemesthetic sensations. The training also included reaching an agreement on the evaluation protocol within the panel, using references to evaluate aroma attributes and numbing sensation as a function of exposure time and familiarising the panel with the intensity scale for rating their perceived intensity of sensory descriptors.

To prepare the samples for aroma evaluation, ten whole Hui Li red Sichuan peppers were randomly selected from each harvest time and placed into 5 mL odourless, transparent glass vials with lids, each labelled with a random three-digit code. The samples were served in a randomised balanced design. During the formal aroma evaluation, the panel was instructed to sniff each sample and rate the intensity for each aroma attribute using relevant reference compounds: hexanol (referred as green aroma), sanshool (referred as Sichuan pepper numbing aroma), 4-ethylguaiacol (referred as woody aroma), nerol (referred as sweet aroma), menthol (referred as fresh aroma), and D-limonene (referred as citrus aroma), on a scale from 0 (very weak) to 9 (very strong). A 1 min interval was maintained between samples to allow the panel to rest and clear their noses before proceeding to the next sample.

To prepare the samples for taste, flavour and chemesthetic sensations evaluation, Hui Li red Sichuan peppers from each harvest time were deseeded and soaked in boiling water for 3 min at a seed-to-water ratio of 1:100 (*w*/*v*). The peppers were then removed, and the remaining Sichuan pepper water extracts were cooled to room temperature and used for sensory evaluation within 2 h. At the start of the formal evaluation, panellists were instructed to rinse their mouths with filtered drinking water. Then, 5 mL of each Sichuan pepper water extract (presented to panellist in randomised order) was delivered to the panellist. The panellists were instructed to taste the Sichuan pepper water extracts and record their perceived intensity of taste (sweetness, saltiness, umami, bitterness), flavour (citrus, lingering), and chemesthetic sensations (cooling, spiciness), on a scale from 0 (very weak) to 9 (very strong). A 30 min break was given between the samples. The panellists were provided with warm purified water and salt-free crackers for palate cleansing.

Furthermore, the Hui Li red Sichuan pepper water extracts from each harvest time were also evaluated by the panellists using the same abovementioned protocol to rate their numbing intensity as a function of exposure time over 900 s. The numbing intensity evaluation began when 150 μL of the Sichuan pepper water extract was introduced to the anterior part of the tongue, with perceived numbness recorded every 10 s for the first 90 s, and then every 30 s thereafter, up to a total duration of 900 s. The panellists rated the numbness on a 10 cm line scale where 0 cm on the scale indicated “no numbness”, 3 cm indicated “slight numbness”, 7.5 cm indicated “moderate numbness”, and 10 cm indicated “strong numbness”. A 30 min break was given between the samples. The panellists were provided with warm purified water and salt-free crackers for palate cleansing.

### 2.3. Overall Aroma Profile Analysis of Hui Li Red Sichuan Peppers Using E-Nose

A Fox 4000 electronic nose (Alpha M.O.S., Toulouse, France), equipped with 18 metal-oxide sensors, was employed to obtain the digitalised odour fingerprint of Sichuan peppers harvested at five different harvest periods. The sensitivities of those E-nose sensors were as follows: PA/2, LY2/AA, P30/2, P30/1, and TA/2 were sensitive to organic compounds; LY2/LG, P40/1, P40/2, T40/2, and T40/1 were sensitive to strong oxidising gases; LY2/G, LY2/Gh, and LY2/g CTl were sensitive to organic amines; P10/1, LY2/gCT, and P10/2 were sensitive to hydrocarbons; T30/1 was sensitive to polar compounds; and T70/2 was sensitive to aromatic compounds [22].

Whole Hui Li red Sichuan peppers from each harvest time were ground, and 0.2 g of the ground material was accurately weighed and transferred into a 10-mL headspace vial for the E-nose analysis with the following operating settings: equilibration at 70 °C for 5 min, injection temperature of 70 °C, injection speed of 500 μL/s, injection period of 1 s, data acquisition for 120 s, and a detection time of 180 s. Each sample was tested in parallel for ten times, and the data from the three most consistent sensor readings were selected for data analysis.

### 2.4. Volatile Compositional Analysis of Hui Li Red Sichuan Peppers Using Headspace Gas Chromatography-Ion Mobility Spectrometry (HS-GC-IMS)

Approximately 0.4 g of the Hui Li red Sichuan peppers from each harvest time was placed into a 20-mL headspace vial and then analysed using a static headspace sampling method with an automatic sampler unit (CTC Analytics AG, Zwingen, Switzerland), followed by GC-IMS analysis (FlavourSpec, G.A.S., Dortmund, Germany). After 10 min of incubation at 60 °C, 500 μL of the headspace content was automatically injected into GC-IMS with a heated syringe (85 °C). Chromatographic separation was performed on a MXT-5 capillary column (15 m × 0.53 mm) with nitrogen (N2, purity ≥99.999%) as the carrier gas at an initial flow rate of 2 mL/min for 2 min, then linearly increased to 10 mL/min for 3 min, followed by an increase to 15 mL/min for 10 min, 50 mL/min for 5 min, and finally 100 mL/min for 10 min. Each sample was analysed in triplicate. GC-IMS data were subjected to qualitative analysis using the instrument built-in databases (NIST and IMS 2020) to assist identification of individual volatile compounds. Regarding the quantitative analysis of volatile compounds, it mainly depended on the peak intensity detected in the HS-GC-IMS, which is directly proportional to the abundance of the volatile compound. GC-IMS fingerprint spectra analysis was conducted using the Gallery plug-in of the software (G.A.S., Dortmund, Germany).

### 2.5. Calculation of Relative Odour Activity Value (ROAV) for Individual Volatiles Identified in Hui Li Red Sichuan Peppers

*ROAV* was used to evaluate the contribution of each volatile compound detected by GC-IMS to the overall aroma of Hui Li red Sichuan peppers from each harvest time. Typically, an *ROAV* > 1 indicates that a volatile compound is a characteristic contributor to the overall aroma. If the *ROAV* is between 0.1 and 1, the volatile compound is considered to have a modifying effect on the overall aroma. Volatile compounds with an *ROAV* < 0.1 are considered as minor aroma contributors [23].

$$ROAV=\frac{C_{i}}{T_{i}}\times \frac{T_{max}}{C_{max}}\times 100$$

where *C_i_* is the relative content of the volatile compound of interest, *C_max_* represents the relative content of the volatile compound with the highest odour activity value among all detected volatiles, *T_i_* is the aroma threshold value of the volatile compound of interest, and *T_max_* represents the aroma threshold value of the volatile compound with the highest odour activity value among all detected volatiles [24].

### 2.6. Data Analysis

Univariate analysis of variance (ANOVA) was performed using SPSS 25.0 Statistics (IBM Corp., Armonk, NY, USA), and Duncan’s multiple comparison test was used to assess differences in the volatile compositions among samples, with a significance level of *p* ≤ 0.05. An electronic nose radar map and principal component (PCA) analyses were generated using Origin 2022 (OriginLab Corporation, Northampton, MA, USA). Partial least squares discriminant analysis (PLS-DA) and VIP were carried out using SIMCA 14.1 software (Umetrics, Umea, Sweden). The two-dimensional coordinate loading plot of principal components was drawn with the samples of different harvest periods as the independent variable (X) and the ROAV values (>0.1) of each volatile substance as the dependent variable (Y). PLS-DA biplots were generated using GraphPad Prism version 8.3.0 (GraphPad Software Inc., San Diego, CA, USA) and Origin 2023b (OriginLab Inc., Northampton, MA, USA).

## 3. Results and Discussion

### 3.1. Sensory Profile of Hui Li Red Sichuan Peppers Across Harvesting Periods

**Figure 1:**
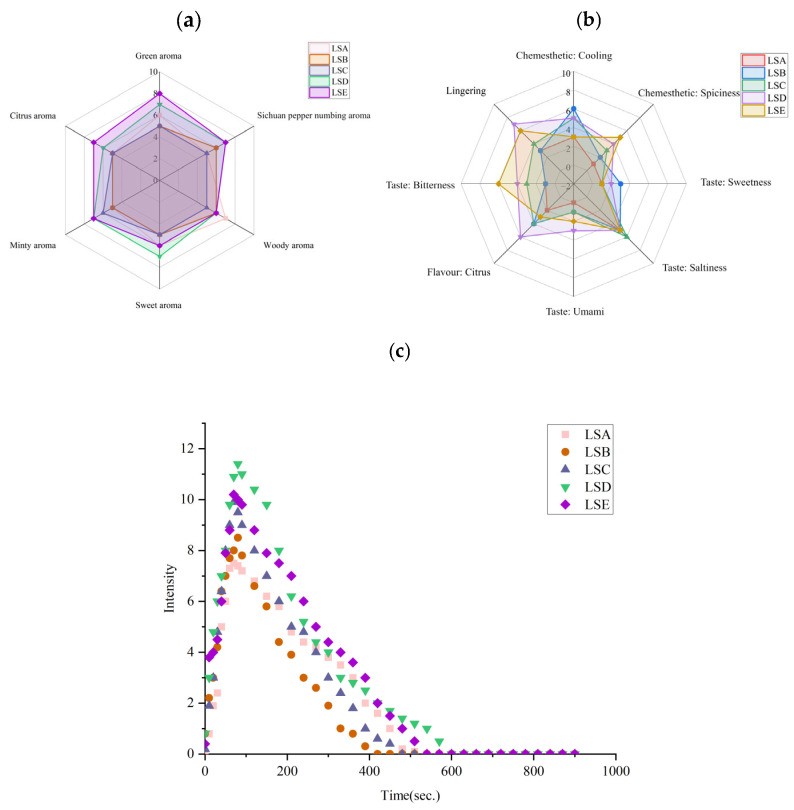
Sensory analysis results from 10 trained panellists on selected (**a**) aroma attributes, (**b**) taste, lingering, flavour attributes, chemesthetic sensations and (**c**) numbing intensity of Hui Li red Sichuan peppers harvested at five selected time periods.

The key aroma attributes of whole Hui Li red Sichuan peppers were defined by 10 trained panellists as green, citrus, minty, sweet, woody, and Sichuan pepper numbing. The perceived intensity of these aroma attributes for pepper samples is illustrated in Figure 1a. Specifically, LSA (late July harvest) sample received the highest perceived intensity for woody aroma, the LSD (early September harvest) sample was characterised by a higher perceived intensity for sweet aroma, and LSE (late September harvest) exhibited higher perceived intensities for green, citrus and fresh aroma. Both minty and pepper-numbing aromas were equally prominent in the LSD and LSE samples as they received the highest intensity ratings compared to those pepper samples harvested before September. Clearly, the variations in aroma attributes across harvest times for whole Hui Li red Sichuan peppers suggest that differences in maturity levels may influence the biosynthesis of volatile compounds, as developmental changes in peppers can affect the pathways responsible for aroma compounds production [25].

Water extracts from whole Hui Li red Sichuan peppers were evaluated by the same trained panellists for their perceived intensity levels of taste, flavour and chemesthetic sensations. The perceived intensities for eight selected attributes (sweetness, saltiness, bitterness, umami taste, citrus flavour, as well as cooling, spiciness and lingering sensations) were found to differ significantly across pepper samples (Figure 1b). Specifically, the water extract from the LSA sample was lacking in all attributes, receiving the lowest perceived intensity ratings across all harvest periods. This suggests that harvesting the pepper in late July may not be ideal for developing key sensory characteristics, likely due to incomplete maturation affecting the accumulation of key flavour compounds. In contrast, the water extract from the LSB sample was rated high in cooling sensation and sweetness but rated lower in umami and bitterness taste attributes, as well as both spiciness and lingering sensations. This indicates that peppers harvested in early August may develop certain desirable characteristics but may still be lacking the full complexity of taste and chemesthetic triggers that are unique to Hui Li red Sichuan pepper. The water extract from the LSC sample showed moderate perceived intensity levels across all evaluated attributes, suggesting a more balanced taste, flavour and chemesthetic profile without the pronounced intensities that began to emerge in the later harvests starting in September. The water extract from the LSD sample had the highest perceived intensity for umami taste, citrus flavour, and lingering sensation. This implies that harvesting in early September may be ideal for applications where umami taste and citrus flavour for Hui Li red Sichuan pepper are the most desirable. The water extract from the LSE sample (late September) was rated highest in bitterness taste and spiciness sensation compared to earlier harvests. This is likely due to the accumulation of bitter compounds, such as alkaloids, terpenes, flavonoids, and coumarins, in the Sichuan peppers as the harvest period extended [1]. Furthermore, perceived spiciness in Sichuan pepper strongly correlates with sanshool content [26], suggesting that sanshool accumulation is highly dependent on the maturation of Sichuan pepper, thus highlighting the importance of harvest timing for Sichuan pepper to maximise the spiciness intensity in Sichuan pepper. The perceived intensity of saltiness, however, remained relatively consistent across all harvest periods of Hui Li red Sichuan peppers, indicating that this taste attribute is less influenced by harvest timing.

Basic human tastes on sourness, sweetness, bitterness, saltiness, and umami are characterised by rapid perception occurring within short duration. In contrast, numbing is a chemesthetic sensation that is slower to perceive, lasts longer, and its intensity changes dynamically over time [27]. The numbing sensation in Sichuan pepper is primarily initiated by unsaturated amides, primarily sanshool compounds and the varying intensity of this sensation is attributed to the distinct interactions of sanshools with membrane ion channels, including TRPV1, TRPA1, KCNK [28]. As shown in Figure 1c, the numbing sensation of water extracts from whole Hui Li red Sichuan pepper rapidly increased upon exposure, reaching a peak intensity between 50 and 180 s, and gradually decreased as exposure time progressed, with the numbing sensation dissipating between 420 and 600 s. Water extracts from all Sichuan pepper samples across the harvesting periods exhibited a rapid increase in perceived numbing intensity, followed by a gradual decline. The perceived numbing intensity, ranked from highest to lowest was LSD > LSE > LSC > LSB > LSA, indicating that Sichuan pepper water extracts with the strongest numbing intensity led to a longer experience duration of the sensation after exposure.

The numbing chemesthetic sensation is an important quality aspect for Sichuan peppers. It has been reported that the chemical compounds responsible for the numbing sensation in red Sichuan pepper, such as hydroxy-α-sanshool, hydroxy-β-sanshool and hydroxy-γ-sanshool, accumulate in large quantities in the leaves during the early stages of growth and maturity, and as the pepper continues to grow, these compounds are gradually transported to the fruit pericarp, resulting in an increased numbing sensation as the peppers mature [25]. Additionally, it has been recognised that the growth environment can play a significant role in the accumulation of numbing compounds, with Sichuan peppers grown in high-altitude areas with consistent and longer sunlight exposure accumulating more amide compounds compared to those grown in lower-altitude regions with limited sunlight exposure [29]. Hui Li red Sichuan peppers, cultivated in high-altitude and sunny regions, are expected to benefit from these environmental conditions, leading to a higher accumulation of numbing compounds. In this study, the most intense perceived numbing sensation was observed in peppers harvested in early September (LSD), likely owing to the continuous increase in sanshool synthesis during the early stages. By late September, the sanshool content may have decreased slightly LSE peppers, possibly due to increased rainfall during that period (Table 1). Overall, findings from the sensory evaluation in this study by a trained panel revealed that Hui Li red Sichuan peppers harvested between early and late September or between White Dew and Autumn Equinox according to the 24 solar terms (i.e., LSD and LSE samples) exhibited a longer-lasting numbing sensation after exposure, as well as stronger perceived green, citrus, minty, sweet and pepper-numbing aroma, along with enhanced umami, bitterness taste, citrus flavour, and lingering, spiciness and numbing chemesthetic sensations, which were lacking in peppers harvested earlier than this time period.

### 3.2. Characterisation of the Overall Aroma Profile of Hui Li Red Sichuan Peppers Across Harvesting Periods

**Figure 2:**
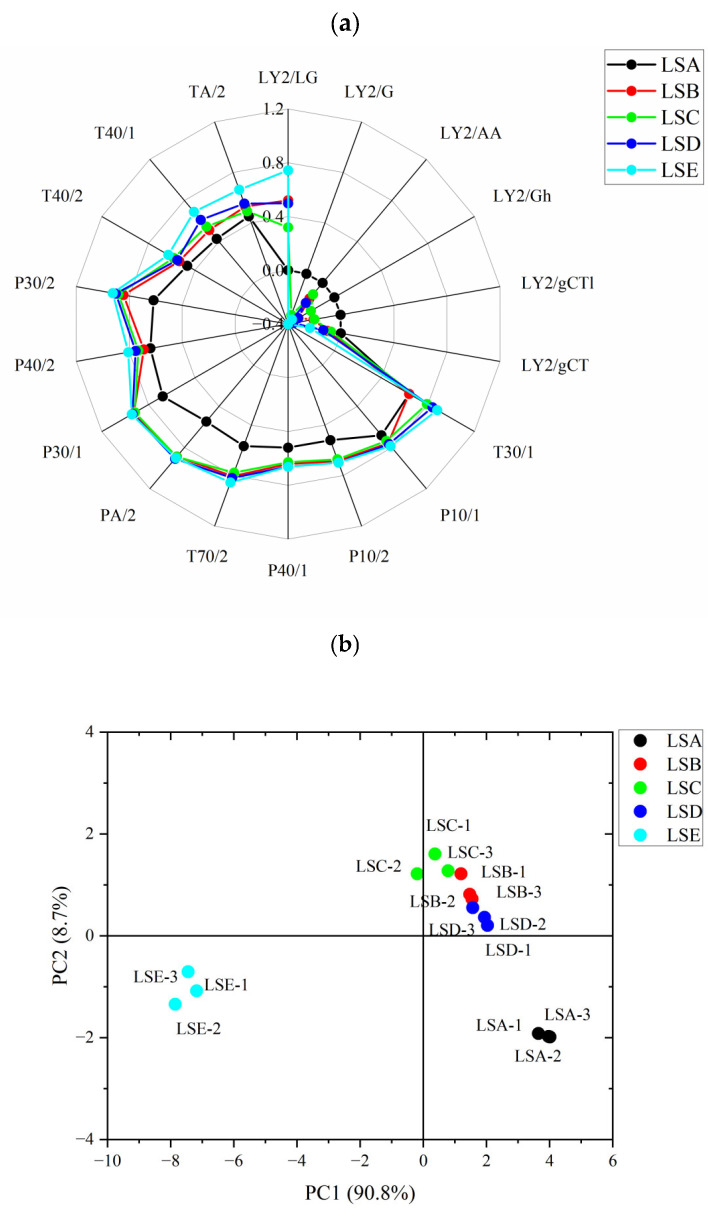
(**a**) E-nose signal radar chart for the different sensors and (**b**) PCA score plot based on e-nose signals of Hui Li red Sichuan peppers harvested at five selected time periods.

E-nose has been used widely employed as an analytical tool for food quality control purpose, with sensors simulating the human olfactory system and exhibiting high sensitivity capable of detecting a wide range of aromatic volatile compounds, thus enabling the identification of even minor variations in these compounds [30]. In this study, an E-nose was used to distinguish the overall aroma profile of the headspace of Hui Li red Sichuan peppers. Figure 2a shows distinct E-nose sensor response values, highlighting variations in the volatile characteristics of Sichuan peppers, which further suggests notable differences in the intensities and proportions of volatile compounds present in each harvest time point. In particular, there were differences in the sensor response values for LY2/LG, TA/2, T40/1, T40/2, P30/2, P40/2, P30/1, PA/2, T70/2, P40/1, P10/2, P10/1, and T30/1. The LSA sample (late July harvest) exhibited the lowest sensor response values compared to other harvest times, indicating that its aroma was less intense and milder than that of peppers harvested at later stages. This could be attributed to the LSA samples being in the early stages of maturity, with incomplete synthesis of volatile oils in the pepper pericarp (Feng et al., 2024 [31]). In contrast, the LSE sample (late September harvest) exhibited higher responses from the LY2/LG, TA/2, T40/1, T40/2, P30/2, and P40/2 sensors, which are more sensitive to oxidising and organic compounds, such as α-terpineol and hexanol in Sichuan peppers [22]. Result from the E-nose analysis suggests that LSE peppers contained a higher concentration of aromatic volatile compounds with strong oxidising properties.

To further visualise the differences in the overall aroma profile among Hui Li red Sichuan pepper samples harvested at different times, principal component analysis (PCA) was conducted on the E-nose response values from the (Figure 2b). The first principal component (PC1) accounted for 86% of the variance, while the second principal component (PC2) accounted for 7.8%, resulting in a cumulative contribution rate exceeding 93%. This indicates that the two PCs captured the majority of the variance in the aroma profiles, effectively differentiating the Sichuan pepper samples based on their volatile compound characteristics detected by the E-nose. The LSA samples formed one distinct cluster, while the LSE samples formed another separated by PC1. The LSB, LSC, and LSD samples clustered together in between the LSA and LSE samples. This demonstrates that the effectiveness of E-nose in distinguishing Hui Li red Sichuan peppers harvested at different times. The large separation between LSA and LSE samples on the PC1 axis indicates significant differences in the aroma profiles of peppers harvested two months apart, likely due to the occurrence of continuous synthesis and maturity of volatile compounds [31]. In contrast, the close proximity of the LSB, LSC and LSD samples suggests a high similarity in their volatile compounds across these harvest periods, with only minor differences observed. This implies that harvesting Hui Li red Sichuan peppers during these stages (i.e., between Beginning of Autumn and White Dew according to the 24 solar terms) will likely result in similar aroma characteristics. It is important to acknowledge that while E-nose is effective in detecting overall aroma differences between the pepper samples, E-nose is limited in its ability to perform qualitative and quantitative analyses of specific volatile aromatic compounds detected from the samples. As a result, the observed large aroma variations between the LSA and LSE samples, as well as the minor aroma differences among the LSB, LSC and LSD samples, cannot be precisely linked to specific volatile compounds responsible for the observed aroma differences. To achieve a comprehensive understanding of the biochemical changes affecting the aroma of Hui Li red Sichuan peppers, the subsequent use of GC-based volatile analysis (Section 3.3) in this study would be helpful in identifying and quantifying individual volatile compounds, providing insights that can explain the findings obtained from the E-nose.

### 3.3. Identification and Quantification of Volatiles in Hui Li Red Sichuan Peppers Across Harvesting Periods

**Figure 3:**
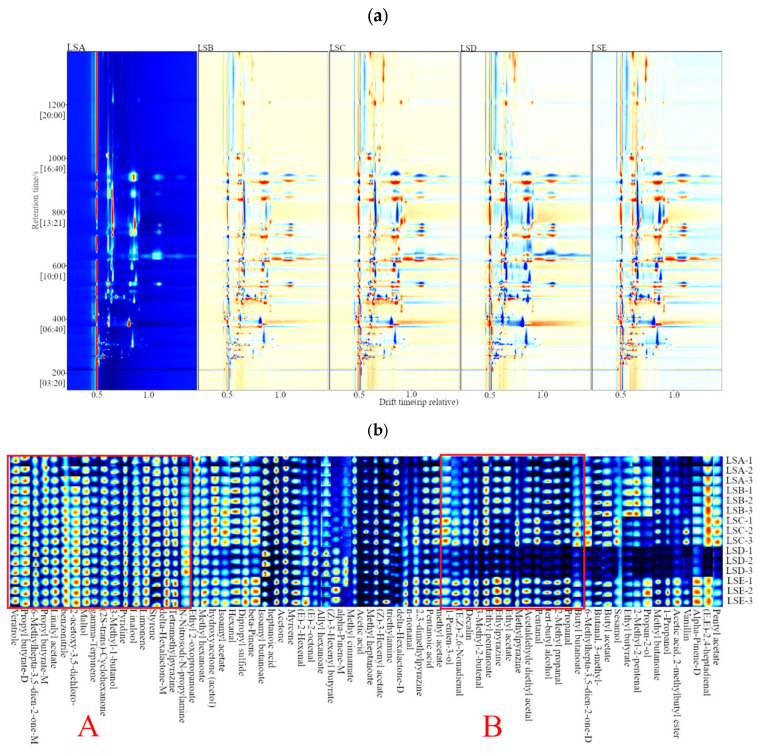
Representative GC-IMS plots of individual volatile compounds detected in Hui Li red Sichuan peppers harvested at five selected time periods: (**a**) 2D topography plot with differential comparison mode; (**b**) gallery plot of signal peak areas of individual volatile compounds. Volatiles in region A consistently exhibited high abundance across all harvesting times, indicating they were not affected by the harvest time. Volatiles in region B showed an increasing trend in abundance during the first three harvest periods (LSA, LSB, and LSC samples, from late July to late August 2022), followed by a sharp decrease at the 4th harvest (LSD sample, early September 2022) and a subsequent increase at the 5th harvest (LSE sample, late September 2022).

GC-IMS is a qualitative method that relies on chemical identities to recognise analytes and is rooted in the two-dimensional separation of component analytes [32]. As shown in Figure 3a, the *x*-axis represents drift time (ms), whereas the *y*-axis represents retention time (s). The red vertical lines on the left side indicate the reactive ion peaks, and the blue colour represents the background of the image. Each dot in Figure 3a represents a different volatile compound, and different shades in the spectrum indicate different concentrations: white dots denote lower concentrations, whereas red dots indicate higher concentrations. Thus, the darker the shade, the higher the concentration. Using the LSA sample as a reference, variations in volatile compounds among samples could be clearly observed. Blue dots represent compounds with high concentrations in the LSA sample, and red dots indicate higher concentrations of these compounds in the samples [33].

Based on the volatile fingerprint map capturing the qualitative and quantitative differences in the volatile organic compounds present in the Hui Li red Sichuan pepper samples (Figure 3b), two distinct trends in volatile composition, represented by regions A and B, emerged as the harvest period of the peppers was extended. In region A, volatiles from the terpenes, alcohols, and esters chemical classes, particularly γ-terpinene, δ-hexalactone-M, styrene, dipentene, linalool, isoamyl alcohol, linalyl acetate, propyl butyrate-M, and propyl butyrate-D, were found in consistently high concentrations across all five selected harvest periods, with only minor fluctuations in concentration over the harvest periods. Since linalool has been reported as a major volatile component of Sichuan pepper [34], linalool showed minimal variation in concentration across the harvest periods, which is consistent with previous studies, confirming the appropriateness of the selected harvest times for this study. In contrast, region B in Figure 3b represents volatile compounds in Hui Li red Sichuan peppers that exhibited significant variation in concentrations between harvest periods where their concentration increased during the early harvest, sharply decreased in the LSD sample (early September harvest) and then rose again in the LSE sample (late September harvest). For example, volatiles such as butylacetate; 3-methylbutanal, 6-methylhepta-3,5-dien-2-one-D, butyl butyrate, propanal, isobutyraldehyde, tert-butanol, valeraldehyde, 2-methylpyrazine, ethyl acetate, 2-ethylpyrazine, ethyl valerate, 3-methyl-2-butenal, (E, Z)-2,6-nonadienal, and 1-penten-3-ol were found in lower abundance in LSD sample. These volatile compounds are known to be mainly concentrated in uneven oil sacs on the surface of the pepper pericarp, where previous research by Huang et al. [1] has indicated that the primary volatile components of Sichuan pepper include a wide range of volatile oils, such as monoterpenes, alcohols, ketones, aldehydes, esters, and epoxides, which can fluctuate depending on the maturity status of the peppers and changing environmental conditions prior to harvest. Specifically in the present study, such fluctuation may be attributed to the ongoing synthesis and accumulation of volatile compounds in Sichuan peppers from mid-July to late August (LSA–LSC samples). However, the significant increases in average ground wind speed (Table 1) may have resulted in a substantial loss of volatile compounds from the surface of the pepper pericarp, thereby reducing their concentration within the LSD sample. As it progressed into the LSE period, the average ground wind speed decreased considerably, coupled with a decline in average temperature, leading to a resurgence in the content of these volatile compounds.

A total of 71 volatile compounds were identified across the Hui Li red Sichuan peppers harvest periods (Table 2), consisting of 5 pyrazines, 6 alcohols, 12 aldehydes, 3 acids, 6 terpenes, 6 ketones, 23 esters, and 10 from other chemical classes (Supplementary Material Figure S2). Esters were the most abundant class of volatile compounds found in the Sichuan peppers in both quantity and concentration, with their levels generally increasing throughout the growth period (late July to late August harvest), declining in the LSD sample (early September harvest), and peaking in the LSE sample (late September harvest). The trend observed with esters is consistent with the E-nose response values (Figure 3a), further supporting the observed changes in the volatile profile of samples. These esters, synthesised primarily through esterification during the maturation of Sichuan peppers, accumulated as the pepper ripened, thus contributing to its rich fruity aroma [35]. In this study, a significant (*p* < 0.05) higher levels of (Z)-3-hexenyl acetate, delta-hexalactone, butyl butanoate, ethyl pentanoate, linalyl acetate, methyl cinnamate, methyl hexanoate and propyl butyrate-D (monomer and dimer) were detected in samples (Table 2). Among these, linalyl acetate showed significant variation (*p* < 0.05), with its concentration increasing steadily as the harvest progressed, reaching its highest level in the LSE phase. Being a major component of the volatile oil in red Sichuan pepper, linalyl acetate accounts for approximately 15% of the total volatile oil content and is considered an important marker for evaluating the quality and maturity of Sichuan pepper [13,34,36]. Therefore, this result suggests that Sichuan pepper harvested in the LSE period were of higher quality and maturity. In contrast, delta-hexalactone was the most abundant in the LSA phase, but decreased as the harvest period extended, indicating it degraded with maturation, allowing for the formation of other more potent volatile compounds. Meanwhile, changes in the levels of methyl cinnamate, methyl hexanoate and propyl butyrate were not statistically significant (*p* > 0.05) throughout the maturation process of Sichuan pepper, indicating that harvest time had minimal impact on these specific volatile compounds.

Terpenes are typical key volatile compounds in spices with low odour thresholds with the molecular formula (C5H8)n, where n represents the number of isoprene unitsand are considered as metabolic products in plants [37]. These compounds are predominantly synthesised via the mevalonate and 2-C-methyl-D-erythritol-4-phosphate pathways, in which the latter is also known as the 1-deoxy-D-xylulose-5-phosphate pathway [38,39]. In this study, the concentration of most terpene compounds in Sichuan peppers, including 2-pinene-D, β-pinene, terpinene, limonene, and myrcene, increased as the harvest period extended, peaking during the LSD and LSE stages (early to late September) (Table 2). This trend is consistent with previous research [25] in which linalyl acetate, linalool and limonene were the claimed to be major aroma components of the leaves and pericarps of *Z. bungeanum*. Since terpinene, limonene and myrcene are widely recognised as characteristic volatile compounds in Sichuan peppers [40], the result observed in the present study suggests that the optimal harvest period for Hui Li red Sichuan pepper is during the LSD and LSE stages, when the levels of these volatiles at their highest. These findings are also in line with previous sensory analysis results (Figure 1a), further reinforcing the period between White Dew and Autumn Equinox, according to the 24 solar terms, as the ideal harvest time for maximising the aroma quality of Hui Li red Sichuan peppers.

Aldehyde compounds were also identified as significant volatiles of Hui Li red Sichuan peppers, with their total content initially increasing and then decreasing as the harvest period extended, peaking at the LSE stage (Table 2). Specifically, 2-hexenal (E), (E, E)-2,4-heptadienal and *n*-nonanal remained at consistently high concentrations throughout the maturation, although their levels fluctuated over time as the harvest period progressed. This pattern suggests that these aldehydes play a key role in shaping the aroma profile of Sichuan pepper, with their dynamics closely tied to their maturity status prior to harvest. On the other hand, the concentration of alcohol compounds showed minimal variation across the different harvest periods (Table 2), indicating that the timing of harvest had a limited impact on the total alcohol content of Hui Li red Sichuan peppers. Linalool and 3-methyl-1-butanol both emerged as the primary alcohols in the pepper samples, maintaining consistently high concentrations throughout the harvest period, particularly between the White Dew and Autumn Equinox periods, as observed in both LSD and LSE samples. Given that linalool is a key aroma compound in Sichuan peppers, its sustained presence during these late harvest stages suggests that peppers harvested during this period are characterised with rich aroma, making them optimal for harvest.

### 3.4. Evaluation of the Contribution of Individual Volatiles in Hui Li Red Sichuan Peppers Across Harvesting Periods

Not all volatile compounds contribute equally to the overall aroma of a food product, as their contributions depend on their odour threshold values and concentrations [41]. Therefore, the relative odour activity value (ROAV) was calculated to evaluate the contribution of each volatile compound identified with GC-IMS to the overall aroma profile of Hui Li red Sichuan peppers. As indicated in Table 3, linalool, characterised with a citrus aroma and an odour threshold of 0.00022 mg/kg, contributed the highest ROAV, set at 100, identifying it as the most impact volatile compound for Hui Li red Sichuan pepper. Volatiles with ROAV between 0 and 1 are considered to exert a modifying effect on the aroma profile, whereas those with ROAV exceeding 1 significantly contribute to the overall aroma of the Sichuan peppers.

Table 3 reveals that compounds such as (E, Z)-2,6-nonadienal, 3-methyl-butanal, ethyl butyrate, ethyl pentanoate, isoamyl acetate, linalool, *n*-nonanal and pentanoic acid maintained ROAV > 1 across all harvest times in Hui Li red Sichuan peppers, indicating their significant contribution to their overall aroma profile. Despite this, there were several key volatile compounds that varied across harvest periods, making unique contributions that characterise the distinct aroma profile of Sichuan pepper at specific harvest times. Esters such as ethylbutyrate (pineapple-like) and isoamyl acetate (banana-like) had high ROAV in the LSC sample (10.51 and 9.10, respectively), contributing rich tropical fruity notes to mid-harvest peppers (late August or End of Heat), while ethyl pentanoate (apple-like) peaked in the LSE peppers (late September or Autumn Equinox), indicating a shift toward ester-dominant aromas in later harvests. Pentanoic acid, with its unpleasant odour characteristic, showed a high ROAV in the LSC sample (8.37), potentially affecting the aroma negatively during this harvest period. Aldehydes with low odour thresholds also played a crucial role; for instance, *n*-nonanal (green and slightly sweet) had a peak ROAV in the LSA sample (late July harvest or Greater Heat), while 3-methyl-butanal (apple-like) was the highest in the LSB sample, suggesting an enhanced fruitiness aroma note from early-to-mid harvest peppers. (E, Z)-2,6-nonadienal, known for its violet and cucumber-like aroma, exhibited fluctuating ROAV across harvest periods and reached its peak in the LSC sample (26.79), marking mid-harvest as a phase of maximum aromatic impact.

### 3.5. Classification of Key Volatile Compounds in Hui Li Red Sichuan Peppers Across Harvesting Periods Identified Using PLS-DA

**Figure 4:**
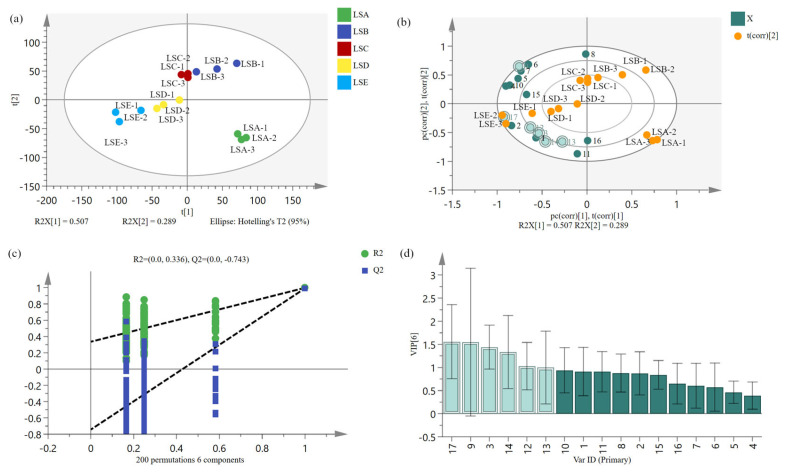
PLS-DA analysis of volatile compounds with calculated ROAV ≥ 1 (refer to Table 3) in Hui Li red Sichuan peppers harvested at five selected time periods. (**a**) Score plot; (**b**) biplot analysis, and where No.1 represents (E)-2-octenal, No.2 represents (E,Z)-2,6-Nonadienal, No.3 represents (Z)-3-Hexenyl acetate, No.4 represents 2-Methyl propanal, No.5 represents acetaldehyde diethyl acetal, No.6 represents Butanal, 3-methyl-, No.7 representsbutyl acetate, No.8 represents Ethyl butyrate, No.9 represents ethyl pentanoate, No.10 represents Isoamyl acetate, No.11 represents Linalool, No.12 represents Methyl heptanoate, No.13 represents Myrcene, No.14 represents *n*-nonanal, No.15 represents Pentanoic acid, No.16 represents triethylamine, No.17 represents Vanillin. Small dark dots represent substances with VIP less than 1, while large light-colored dots represent substances with VIP greater than 1; (**c**) PLS-DA model validation plot; (**d**) VIP score plot, where light blue represents volatile compounds with calculated VIP value > 1 and dark blue represents volatile compounds with calculated VIP value <1; PLS-DA: Partial Least Squares Discriminant Analysis; ROAV: Relative Odour Activity Value; VIP: Variable Importance in Projection.

PLS-DA, as a supervised multivariate statistical analysis method, is considered an effective model for biological data and provides a powerful discrimination method for samples with different chemical characteristics [42,43]. In this study, the PLS-DA model was employed, focusing on volatile compounds (identified earlier with GC-IMS) with ROAV > 1, to identify specific marker compounds that account for the aroma differentiation in samples. As illustrated in Figure 4a, the model yielded R2X1 = 0.507 and R2X2 = 0.289, with a cumulative contribution exceeding 75%, indicating high model reliability [44]. Based on their volatile composition, the samples were clustered into three distinct groups: LSA (late July harvest) formed one group; LSB, LSC and LSD (harvested between early August and early September) formed a second group; and LSE (late September harvest) formed the third group. The pronounced separation between LSA and LSE in the PLS-DA model suggests significant differences in their volatile composition, whereas the close proximity of LSB, LSC and LSD indicates similar volatile composition among these samples. This finding suggests that the volatile compounds of Hui Li red Sichuan peppers varies with harvest timing, with marked differences in aroma observed between peppers harvested in July and those harvested in September. It is important to note that the separation of Sichuan pepper samples observed in the supervised PLS-DA approach based on volatile composition analysed with GC-IMS (Figure 4a) aligns with the unsupervised PCA result based on the E-nose response signals (Figure 2b). Such consistency clearly implies that harvest timing over two months significantly influences the aroma characteristics of Hui Li red Sichuan peppers, regardless of whether the volatile compounds were detected using E-nose or GC-IMS analytical tools.

In the PLS-DA biplot (Figure 4b), LSA peppers were positively correlated with PC1, whereas LSE and LSD peppers showed negative correlation with PC1. As the harvest period progressed, characteristic volatile compounds in Sichuan peppers were primarily concentrated near the LSE sample. This indicated that these key volatile compounds (with VIP > 1) were more abundant in the LSE sample, suggesting that Sichuan peppers harvested during this period may have a richer aroma profile. The validity of the PLS-DA model was confirmed by 200 cross-validations, where an R2 slope > 0 and a Q2 intercept < 0 indicated that the model did not overfit (Figure 4c). The VIP score assessed the impact and explanatory power of each variable (i.e., volatile compound) on the classification and discrimination of the Hui Li red Sichuan peppers across the harvesting periods. Higher VIP values imply greater differences in volatile compounds between sample groups and greater significance in the discrimination of Sichuan pepper volatiles [45,46]. VIP analysis identified six volatiles with VIP > 1, including (Z)-3-hexenylacetate (No.3), ethyl pentanoate (No.9), methyl heptanoate (No.12), myrcene (No.13), *n*-nonanal (No.14), and vanillin (No.17) (Figure 4d). These compounds are considered characteristic volatiles in Sichuan peppers and contribute significantly to their aroma. Notably, vanillin, which had the highest VIP value, made significant contribution to the overall aroma of the peppers across harvest periods. Additionally, three esters (i.e., (Z)-3-hexenyl acetate, ethyl pentanoate and methyl heptanoate) provided a rich fruity aroma, likely resulting from esterification reactions between alcohols and acids as the pepper matured [47]. At the same time, this result confirms that the diversity of volatile components of samples is mainly driven by the composition and changes in ester compounds.

## 4. Conclusions

The present study has integrated sensory evaluation by a trained panel, E-nose and GC-IMS techniques with statistical tools (i.e., ROAV and PLS-DA) that successfully revealed the pronounced differences in the aroma of Hui Li red Sichuan peppers across various harvest periods within the 24 traditional solar terms. As the harvest period was extended, sensory scores increased for fresh, woody, sweet, green, citrus, and pepper-numbing aroma attributes. Notably, peppers harvested in the LSD phase (early September) exhibited the strongest numbing sensation, a distinctive characteristic of Hui Li red Sichuan peppers. E-nose analysis indicated that the overall aroma of Sichuan peppers varied with the harvest period, with the most significant differences observed between the late July (LSA) and late September (LSE) harvests. GC-IMS analysis identified 71 volatile compounds, among which esters were the predominant aroma contributors, continuously increasing in content over time. Using ROAV in combination with PLS-DA, six key aroma compounds (with VIP > 1) were identified: (Z)-3-hexenyl acetate, ethyl pentanoate, methyl heptanoate, myrcene, *n*-nonanal and vanillin, in which their concentrations rose substantially with harvest time, marking them as key compounds for differentiating Hui Li red Sichuan peppers harvested at different times. As the intensity of numbing taste of samples in LSD phase (early September) had the highest value and the aroma of Sichuan pepper harvested in LSE phase (mid to late September) is more prominent. It is recommended to harvest the Sichuan pepper in September to obtain a better numbing sensation and aroma. These findings have important implications for Sichuan pepper harvesting practices, indicating that harvest time can be optimised to target specific sensory attributes, allowing producers to cater to different market needs, from milder, sweeter flavours to stronger, spicier profiles,while maximising the application value of Sichuan peppers in the seasoning industry. However, this study is constrained by its geographic scope and single-season, single-year sampling design. Future research should prioritize investigations into varietal adaptations, altitudinal gradients, and annual climatic variations to strengthen the generalizability of these findings.

## Figures and Tables

**Table 1:** Environmental data of Sichuan pepper across five harvesting periods selected in this study within the 24 traditional Chinese solar terms.

Sample ID	Harvesting Time	Solar Term *	Average Highest Temperature (°C)	Average Lowest Temperature (°C)	Average Humidity (%)	Average Rainfall (mm/h)	Average Ground Wind Speed (m/s)
LSA	24 July 2022	Greater Heat	29	19	69.5	0.32	1.44
LSB	7 August 2022	Beginning of Autumn	26	18	81.0	0.53	0.81
LSC	22 August 2022	End of Heat	28	18	74.0	0.31	0.91
LSD	6 September 2022	White Dew	26	17	77.8	0.37	1.23
LSE	21 September 2022	Autumn Equinox	24	17	81.2	0.41	0.82

* The environmental data were obtained from the China Weather Historical Database (https://lishi.tianqi.com/), based on the altitude, latitude and longitude of the Sichuan pepper cultivation location.

**Table 2:** Peak areas of volatile compounds in Hui Li red Sichuan pepper identified and quantified using GC–IMS across five different harvesting periods.

Chemical Classes	Volatile Compounds	RI	Dt [a.u.]	CAS No.	LSA	LSB	LSC	LSD	LSE
Pyrazine	2,3-Dimethylpyrazine	1326.8	1.46205	5910-89-4	492.07 ± 111.39 ^c^	646.39 ± 42.07 ^c^	1555.22 ± 108.47 ^a^	1031.68 ± 1.35 ^b^	1671.06 ± 34.46 ^a^
	2-acetoxy-3,5-dichloro-Benzonitrile	1215.5	1.209	54300-08-2	1136.77 ± 269.33 ^b^	1666.53 ± 80.80 ^a^	1849.41 ± 24.97 ^a^	1898.95 ± 23.73 ^a^	1922.53 ± 15.13 ^a^
	Ethylpyrazine	928.7	1.12929	13925-00-3	808.69 ± 184.69 ^bc^	1107.59 ± 147.60 ^b^	743.20 ± 49.14 ^c^	437.18 ± 3.25 ^d^	2038.48 ± 60.80 ^a^
	Methylpyrazine	1238.4	1.38494	109-08-0	481.77 ± 45.97 ^d^	565.06 ± 19.71 ^c^	1028.64 ± 22.21 ^a^	339.19 ± 16.52 ^e^	894.24 ± 26.94 ^b^
	Tetramethylpyrazine	1089.5	1.21335	1124-11-4	2427.98 ± 328.66 ^c^	2975.82 ± 112.57 ^ab^	3066.49 ± 80.70 ^ab^	2634.76 ± 136.14 ^bc^	3418.05 ± 52.02 ^a^
Alcohol	1-Penten-3-ol	1162.3	1.33235	616-25-1	247.96 ± 38.77 ^b^	252.16 ± 6.01 ^b^	360.80 ± 9.21 ^a^	158.66 ± 6.10 ^c^	265.58 ± 9.82 ^b^
	1-Propanol	1029.7	1.11077	71-23-8	193.02 ± 60.99 ^b^	263.73 ± 10.92 ^b^	198.70 ± 7.45 ^b^	89.59 ± 4.79 ^c^	388.88 ± 35.70 ^a^
	3-Methyl-1-butanol	1216	1.24667	123-51-3	1151.00 ± 247.32 ^b^	1471.54 ± 46.05 ^ab^	1625.38 ± 18.22 ^a^	1565.47 ± 16.55 ^a^	1661.89 ± 23.43 ^a^
	Linalool	1098	1.21324	78-70-6	2343.38 ± 340.35 ^b^	2704.90 ± 113.07 ^ab^	2990.69 ± 84.09 ^a^	3200.03 ± 62.94 ^a^	2820.90 ± 63.63 ^ab^
	Propan-2-ol	920.5	1.21867	67-63-0	107.40 ± 41.82 ^b^	173.65 ± 13.18 ^a^	59.34 ± 8.48 ^b^	29.01 ± 3.44 ^c^	203.35 ± 25.36 ^a^
	tert-Butyl alcohol	917.3	1.3273	75-65-0	294.75 ± 69.14 ^a^	375.11 ± 29.13 ^a^	201.66 ± 19.79 ^b^	79.90 ± 2.55 ^c^	386.97 ± 13.67 ^a^
Aldehydes	(E)-2-Hexenal	1240.6	1.1871	6728-26-3	1929.77 ± 202.75 ^c^	2232.73 ± 111.11 ^bc^	2532.72 ± 206.00 ^ab^	2261.98 ± 127.80 ^bc^	2950.86 ± 60.26 ^a^
	(E)-2-octenal	1055.8	1.80732	2548-87-0	388.06 ± 116.70 ^b^	547.99 ± 179.00 ^ab^	1163.31 ± 153.82 ^a^	784.88 ± 280.61 ^ab^	1148.84 ± 279.48 ^a^
	(E,E)-2,4-heptadienal	1008.2	1.60923	4313-3-5	3811.54 ± 818.68 ^a^	4904.04 ± 92.19 ^a^	4457.85 ± 17.85 ^a^	312.23 ± 48.52 ^b^	4140.72 ± 132.71 ^a^
	(E, Z)-2,6-Nonadienal	1181.7	1.36543	557-48-2	710.82 ± 112.00 ^c^	906.78 ± 38.01 ^b^	1562.44 ± 45.65 ^a^	868.30 ± 31.56 ^bc^	1541.97 ± 16.56 ^a^
	2-Methyl propanal	811.6	1.28012	78-84-2	201.20 ± 25.79 ^b^	232.77 ± 13.24 ^b^	307.34 ± 12.16 ^a^	75.58 ± 1.36 ^c^	297.34 ± 3.92 ^a^
	2-Methyl-2-pentenal	828	1.1546	623-36-9	308.66 ± 37.44 ^a^	349.24 ± 8.77 ^a^	150.27 ± 14.01 ^c^	17.67 ± 1.61 ^d^	195.48 ± 12.43 ^b^
	3-Methyl-2-butenal	1198.6	1.3768	107-86-8	375.64 ± 48.78 ^c^	488.62 ± 30.91 ^b^	999.51 ± 43.66 ^a^	377.71 ± 29.47 ^bc^	1008.05 ± 53.09 ^a^
	Butanal, 3-methyl-	918.4	1.3951	590-86-3	456.57 ± 124.26 ^a^	569.93 ± 72.66 ^a^	474.51 ± 36.73 ^a^	121.39 ± 3.96 ^b^	494.99 ± 13.28 ^a^
	Hexanal	1085	1.25966	66-25-1	219.34 ± 28.86 ^a^	264.64 ± 18.72 ^a^	283.83 ± 15.32 ^a^	174.46 ± 17.66 ^b^	226.00 ± 15.98 ^a^
	n-Nonanal	1343.1	1.47441	124-19-6	1004.34 ± 206.95 ^c^	1227.10 ± 27.05 ^c^	1926.58 ± 150.33 ^ab^	1752.63 ± 114.02 ^b^	2414.16 ± 43.23 ^a^
	Pentanal	949.8	1.18461	110-62-3	258.47 ± 83.44 ^c^	376.40 ± 28.16 ^bc^	709.78 ± 34.73 ^a^	120.81 ± 4.28 ^d^	503.66 ± 26.28 ^b^
	Propanal	796.7	1.14548	123-38-6	717.88 ± 127.00 ^d^	969.80 ± 22.51 ^c^	1245.93 ± 65.84 ^b^	201.61 ± 7.79 ^e^	1970.02 ± 27.85 ^a^
Acids	Acetic acid	1445.1	1.05985	64-19-7	6494.35 ± 471.92 ^a^	6974.13 ± 155.23 ^a^	6466.99 ± 101.97 ^a^	5938.75 ± 237.68 ^a^	4822.92 ± 266.24 ^b^
	Heptanoic acid	1080.9	1.36966	111-14-8	3716.29 ± 158.43 ^b^	3973.82 ± 60.31 ^b^	3225.14 ± 88.92 ^c^	5079.51 ± 129.81 ^a^	2430.89 ± 71.95 ^d^
	Pentanoic acid	878.6	1.21964	109-52-4	523.50 ± 70.95 ^bc^	671.41 ± 9.98 ^ab^	634.78 ± 42.33 ^ab^	466.09 ± 55.77 ^c^	820.34 ± 12.75 ^a^
Terpene	alpha-Pinene-D	1014.4	1.66667	80-56-8	564.75 ± 186.14 ^b^	1002.97 ± 129.78 ^a^	1107.48 ± 75.33 ^a^	398.42 ± 85.87 ^b^	1567.86 ± 54.99 ^a^
	alpha-Pinene-M	1013.1	1.29761	80-56-8	46.66 ± 12.87 ^c^	87.91 ± 14.87 ^b^	136.28 ± 12.09 ^b^	319.44 ± 37.53 ^a^	302.27 ± 30.27 ^a^
	beta-Pinene	1088.9	1.29488	127-91-3	1199.46 ± 41.53 ^b^	1307.63 ± 91.06 ^b^	1271.82 ± 39.49 ^b^	1062.20 ± 51.39 ^c^	1582.38 ± 38.51 ^a^
	gamma-Terpinene	1227.7	1.21249	99-85-4	6000.54 ± 472.47 ^b^	7137.74 ± 285.16 ^a^	7005.04 ± 166.52 ^a^	7667.60 ± 212.84 ^a^	7898.78 ± 120.07 ^a^
	Limonene	1204.1	1.68643	138-86-3	270.98 ± 52.40 ^bc^	341.52 ± 1.79 ^b^	508.73 ± 16.04 ^a^	234.38 ± 9.30 ^c^	536.00 ± 12.70 ^a^
	Myrcene	1165.2	1.29095	123-35-3	3259.85 ± 525.43 ^a^	3761.18 ± 111.26 ^a^	3884.18 ± 108.33 ^a^	3954.58 ± 74.85 ^a^	3815.07 ± 77.68 ^a^
Ketone	(2S-trans)-Cyclohexanone	1133	1.33977	14073-97-3	236.35 ± 66.91 ^c^	370.77 ± 38.76 ^b^	485.39 ± 14.41 ^a^	428.86 ± 18.67 ^ab^	463.50 ± 19.40 ^ab^
	6-Methylhepta-3,5-dien-2-one-D	1104	1.74493	1604-28-0	1451.81 ± 261.18 ^c^	2315.92 ± 326.69 ^b^	3446.57 ± 277.32 ^a^	578.10 ± 107.00 ^d^	1953.42 ± 137.19 ^bc^
	6-Methylhepta-3,5-dien-2-one-M	1103.8	1.2198	1604-28-0	2932.68 ± 64.24 ^ab^	2781.60 ± 18.20 ^b^	2494.00 ± 47.91 ^c^	3064.61 ± 117.16 ^a^	3072.72 ± 4.92 ^a^
	Acetone	820	1.11433	67-64-1	5574.27 ± 137.70 ^a^	5768.81 ± 42.00 ^a^	4870.00 ± 110.33 ^c^	2701.50 ± 76.56 ^d^	5301.29 ± 98.56 ^b^
	Hydroxyacetone (acetol)	1321.6	1.24585	116-09-6	465.30 ± 61.27 ^b^	542.04 ± 8.11 ^ab^	554.07 ± 36.79 ^ab^	548.33 ± 21.25 ^ab^	648.81 ± 21.34 ^a^
	Maltol	1133.9	1.21541	118-71-8	220.58 ± 116.91 ^b^	397.73 ± 25.79 ^a^	404.18 ± 20.22 ^a^	493.36 ± 14.19 ^a^	441.66 ± 4.29 ^a^
Esters	(Z)-3-Hexenyl acetate	1302.9	1.30144	3681-71-8	3508.72 ± 424.26 ^c^	4300.56 ± 264.78 ^ab^	5128.53 ± 10.14 ^a^	4400.08 ± 227.93 ^ab^	3785.07 ± 111.57 ^bc^
	(Z)-3-Hexenyl butyrate	1226.5	1.43107	16491-36-4	561.38 ± 47.65 ^a^	431.53 ± 28.57 ^b^	389.55 ± 30.15 ^b^	565.51 ± 33.67 ^a^	380.41 ± 7.49 ^b^
	delta-Hexalactone-D	1082.1	1.52085	823-22-3	1583.55 ± 48.56 ^a^	1471.14 ± 90.91 ^ab^	1219.68 ± 124.89 ^b^	714.47 ± 79.41 ^c^	562.22 ± 38.10 ^d^
	delta-Hexalactone-M	1082.2	1.16828	823-22-3	2012.70 ± 154.13 ^a^	1658.86 ± 109.38 ^bc^	1472.42 ± 11.74 ^c^	1868.31 ± 3.41 ^ab^	1256.75 ± 34.18 ^d^
	Acetic acid, 2-methylbutyl ester	880.7	1.28214	624-41-9	36.10 ± 2.18 ^c^	59.20 ± 6.17 ^b^	35.96 ± 3.39 ^c^	28.26 ± 2.92 ^d^	109.59 ± 6.69 ^a^
	Allyl hexanoate	1352.8	1.37688	123-68-2	445.87 ± 23.04 ^b^	483.25 ± 14.19 ^b^	466.85 ± 21.69 ^b^	818.03 ± 62.46 ^a^	480.64 ± 14.71 ^b^
	Butyl acetate	1076.5	1.62471	123-86-4	519.56 ± 170.12 ^a^	716.31 ± 14.11 ^a^	641.21 ± 21.46 ^a^	237.97 ± 20.41 ^b^	572.37 ± 24.94 ^a^
	Butyl butanoate	1182	1.8007	109-21-7	2172.85 ± 241.62 ^b^	2343.12 ± 79.30 ^b^	3407.15 ± 199.19 ^a^	991.94 ± 60.77 ^c^	2372.91 ± 70.13 ^b^
	Ethyl 2-oxopropanoate	1276.9	1.43614	617-35-6	84.53 ± 5.20 ^cd^	81.12 ± 1.59 ^d^	89.67 ± 4.23 ^bc^	145.50 ± 12.29 ^a^	105.92 ± 4.79 ^b^
	Ethyl acetate	918.8	1.08716	141-78-6	345.56 ± 66.20 ^a^	397.54 ± 23.74 ^a^	165.27 ± 17.29 ^b^	140.24 ± 16.56 ^b^	469.41 ± 35.40 ^a^
	Ethyl butyrate	1031.5	1.21088	105-54-4	450.68 ± 87.92 ^a^	616.07 ± 44.58 ^a^	216.39 ± 20.11 ^b^	148.54 ± 25.48 ^c^	292.72 ± 23.48 ^b^
	Ethyl pentanoate	1126	1.27916	539-82-2	4284.61 ± 415.17 ^b^	4938.86 ± 91.81 ^a^	5012.15 ± 110.59 ^a^	1285.56 ± 66.07 ^c^	4421.81 ± 89.88 ^ab^
	Isoamyl acetate	1105.1	1.30586	123-92-2	1774.07 ± 342.14 ^b^	2310.44 ± 57.14 ^ab^	2541.65 ± 37.44 ^a^	1293.93 ± 59.73 ^c^	1897.17 ± 36.61 ^b^
	Isoamyl butanoate	1251.1	1.39868	106-27-4	617.11 ± 77.58 ^a^	619.98 ± 10.23 ^a^	723.80 ± 25.84 ^a^	646.86 ± 18.05 ^a^	647.29 ± 19.20 ^a^
	Linalyl acetate	1240.7	1.21697	115-95-7	3367.72 ± 323.14 ^c^	4045.86 ± 140.25 ^b^	4406.03 ± 57.38 ^ab^	4556.81 ± 137.18 ^ab^	4896.29 ± 39.00 ^a^
	Methyl acetate	813.9	1.2013	79-20-9	190.62 ± 30.01 ^b^	224.07 ± 5.13 ^ab^	270.10 ± 6.72 ^a^	122.78 ± 9.04 ^c^	271.64 ± 3.09 ^a^
	Methyl butanoate	987.7	1.41948	623-42-7	55.25 ± 33.23 ^b^	161.35 ± 45.94 ^a^	156.18 ± 27.04 ^a^	39.76 ± 0.82 ^b^	209.32 ± 9.65 ^a^
	Methyl cinnamate	1378.8	1.89446	103-26-4	1411.93 ± 248.19 ^a^	1620.99 ± 47.94 ^a^	1378.50 ± 238.95 ^a^	2618.29 ± 1243.20 ^a^	1815.42 ± 1240.45 ^a^
	Methyl heptanoate	1300.4	1.79356	106-73-0	982.87 ± 164.89 ^b^	1184.66 ± 59.89 ^b^	1793.84 ± 106.43 ^a^	1226.77 ± 122.30 ^b^	1006.45 ± 45.06 ^b^
	Methyl hexanoate	1172	1.29599	106-70-7	4447.17 ± 308.09 ^a^	3707.24 ± 277.63 ^a^	3979.41 ± 147.06 ^a^	3844.06 ± 254.30 ^a^	3769.97 ± 128.80 ^a^
	Pentyl acetate	1155.5	1.30771	628-63-7	766.79 ± 126.49 ^c^	946.72 ± 20.28 ^b^	1314.03 ± 12.71 ^a^	335.57 ± 18.64 ^d^	805.30 ± 10.53 ^bc^
	Propyl butyrate-D	1136	1.70615	105-66-8	1379.82 ± 204.56 ^a^	1449.18 ± 148.26 ^a^	1373.51 ± 48.68 ^a^	1668.43 ± 10.32 ^a^	1280.36 ± 100.25 ^a^
	Propyl butyrate-M	1135.3	1.28257	105-66-8	1529.34 ± 109.35 ^a^	1512.22 ± 84.96 ^a^	1296.82 ± 29.43 ^b^	1608.38 ± 59.30 ^a^	1260.71 ± 54.70 ^b^
Others	Acetaldehyde diethyl acetal	872	1.12429	105-57-7	159.92 ± 39.18 ^b^	227.36 ± 8.66 ^a^	211.33 ± 6.85 ^ab^	55.13 ± 1.78 ^c^	266.88 ± 3.01 ^a^
	Decalin	1055.2	1.20924	91-17-8	433.69 ± 66.30 ^b^	593.98 ± 70.53 ^ab^	571.92 ± 57.92 ^ab^	288.76 ± 45.91 ^c^	760.87 ± 37.15 ^a^
	Dipropyl sulphide	880.3	1.16491	111-47-7	195.15 ± 20.65 ^a^	229.84 ± 2.44 ^a^	201.85 ± 12.62 ^a^	150.75 ± 5.54 ^b^	230.86 ± 7.59 ^a^
	N-nitroso-di-N-propylamine	1075.8	1.27549	621-64-7	3466.19 ± 235.85 ^a^	3561.43 ± 122.88 ^a^	3554.94 ± 26.76 ^a^	2336.14 ± 182.74 ^b^	3601.32 ± 54.24 ^a^
	Pyridine	1224	1.24361	110-86-1	3343.69 ± 164.53 ^a^	3558.87 ± 23.87 ^a^	3410.57 ± 43.22 ^a^	3512.98 ± 23.05 ^a^	3436.19 ± 92.56 ^a^
	Sesamol	1311.4	1.20692	533-31-3	431.55 ± 125.65 ^d^	665.59 ± 86.66 ^bc^	1068.64 ± 63.23 ^a^	457.78 ± 48.40 ^cd^	862.70 ± 36.62 ^ab^
	Styrene	881.1	1.04766	100-42-5	709.10 ± 35.82 ^b^	766.31 ± 12.48 ^ab^	780.81 ± 16.63 ^a^	783.85 ± 18.68 ^a^	793.71 ± 15.69 ^a^
	Triethylamine	805.3	1.46738	121-44-8	270.23 ± 108.67 ^a^	354.38 ± 113.91 ^a^	446.39 ± 99.38 ^a^	530.59 ± 155.43 ^a^	448.23 ± 183.96 ^a^
	Vanillin	1389.1	1.27221	121-33-5	1325.37 ± 303.60 ^bc^	1658.74 ± 11.99 ^bc^	4906.87 ± 1189.04 ^a^	996.03 ± 377.00 ^c^	3065.24 ± 895.97 ^ab^
	Veratrole	1147.8	1.28211	91-16-7	4041.35 ± 82.16 ^bc^	4256.20 ± 48.56 ^ab^	3983.61 ± 65.91 ^c^	4228.80 ± 129.13 ^ab^	4342.79 ± 51.51 ^a^

Data are presented as mean ± standard deviation (*n* = 2). Mean values with different letters indicate significant differences (*p* < 0.05) in the volatile compound amounts between harvesting periods, as determined by ANOVA followed by Duncan’s multiple comparison test.

**Table 3:** Relative odour activity value (ROAV) analysis of key volatile identified using GC-IMS in Hui Li red Sichuan pepper across five different harvesting periods.

Volatile Compound	Aroma Characteristic	Odour Threshold Value (mg/kg)	CAS No.	ROAV (%)				
				LSA	LSB	LSC	LSD	LSE
(E)-2-octenal	Citrus	0.0027	2548-87-0	2.96	3.46	3.11	0.22	2.65
(E, Z)-2,6-Nonadienal	Intense violet and cucumber	0.00011	557-48-2	13.60	15.68	26.79	14.78	24.19
(Z)-3-Hexenylacetate	Intense green grass	0.009	3681-71-8	0.82	0.91	1.07	0.92	0.73
2-Methylpropanal	Intense irritating odour	0.001	78-84-2	0.65	0.66	0.28	0.03	0.34
Acetaldehyde diethylacetal	-	0.0049	105-57-7	2.80	2.71	2.49	2.27	1.70
3-Methyl-butanal	Apple-like	0.00035	590-86-3	3.09	3.89	3.45	1.27	2.82
Butylacetate	Fruity	0.01	123-86-4	0.46	0.45	0.64	0.19	0.41
Ethylbutyrate	Pineapple	0.0009	105-54-4	10.06	10.44	10.51	2.68	8.48
Ethylpentanoate	Apple	0.00058	539-82-2	2.92	3.63	2.42	1.41	6.07
Isoamylacetate	Banana	0.00015	123-92-2	8.69	7.86	9.10	8.08	7.45
Linalool	Buddha’s hand-like aroma	0.00022	78-70-6	100.00	100.00	100.00	100.00	100.00
Methylheptanoate	-	0.004	106-73-0	0.74	0.77	0.65	1.22	0.78
Myrcene	Sweet orange and balsam	0.0012	123-35-3	0.85	0.90	1.62	0.53	1.29
n-Nonanal	Green and slightly sweet	0.0031	124-19-6	2.37	2.19	2.16	1.41	2.00
Pentanoic acid	Unpleasant	0.00016	109-52-4	3.37	4.47	8.37	1.41	5.43
Triethylamine	Intense ammonia	0.022	121-44-8	0.23	0.26	0.26	0.22	0.27
Vanillin	Vanilla	0.0012	121-33-5	0.49	0.56	0.70	0.83	0.64

“-” indicates that the aroma characteristic of the volatile is not described where all flavour descriptions were obtained from http://www.flavornet.org/flavornet.html (accessed on 28 May 2025).

## Data Availability

The original contributions presented in this study are included in the article/Supplementary Material. Further inquiries can be directed to the corresponding author.

## Supplementary Materials

The following supporting information can be downloaded at: https://www.mdpi.com/article/10.3390/foods14132285/s1, Figure S1: The 24 solar terms.; Figure S2: Classification of volatile compound in Sichuan pepper at different harvest periods based on peak volumes.
