# Supplementary material for: Understanding the Aroma Profiles of Hui Li Red Sichuan Pepper (Zanthoxylum bungeanum Maxim) Across Harvesting Periods Using Sensory Evaluation, E-Nose and GC-IMS Techniques
Source: Foods. 2025 Jun 27;14(13):2285. doi: 10.3390/foods14132285 (PMC12248581; doi:10.3390/foods14132285)
Supplement: Supplementary file 1 [file foods-14-02285-s001.zip › foods-3622077-supplementary.pdf]

SUPPLEMENTARY MATERIAL

Understanding the Aroma Profiles of Hui Li Red Sichuan Pepper (Zanthoxylum bungeanum Maxim) Across Harvesting Periods Using Sensory Evaluation, E-Nose and GC-IMS Techniques

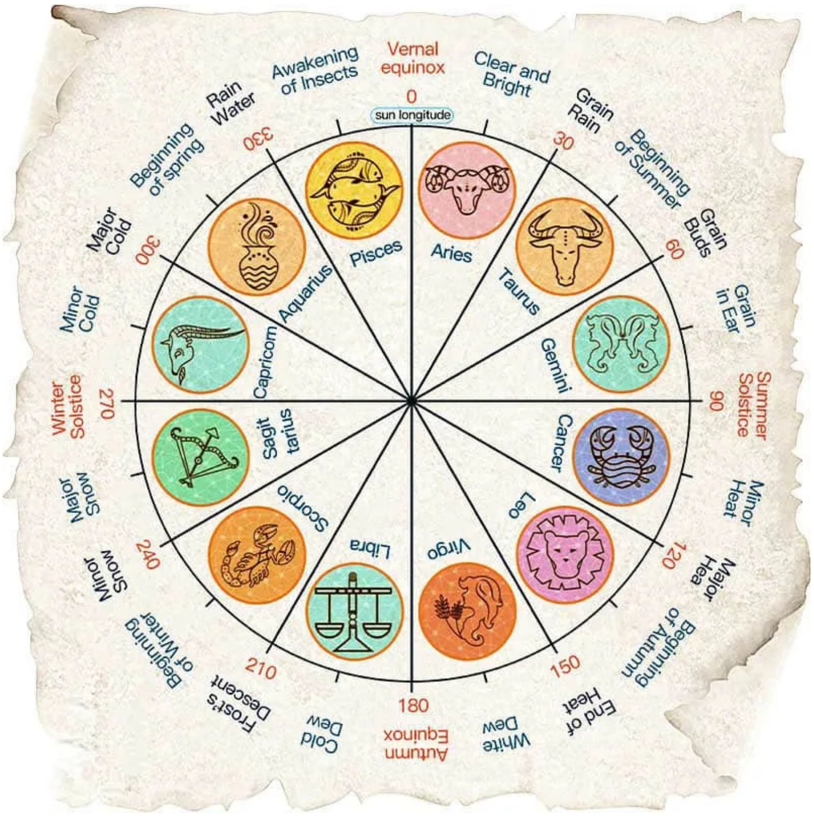

https://www.chinaeducationaltours.com/guide/culture-24-solar-terms.htm

Figure S1. The 24 solar terms.The 24 solar terms are part of the traditional Chinese lunisolar calendar and represent specific natural and seasonal changes throughout the year. Each solar term lasts about 15 days, which guide agricultural activities and mark changes in weather, climate, and natural phenomena.

SUPPLEMENTARY MATERIAL

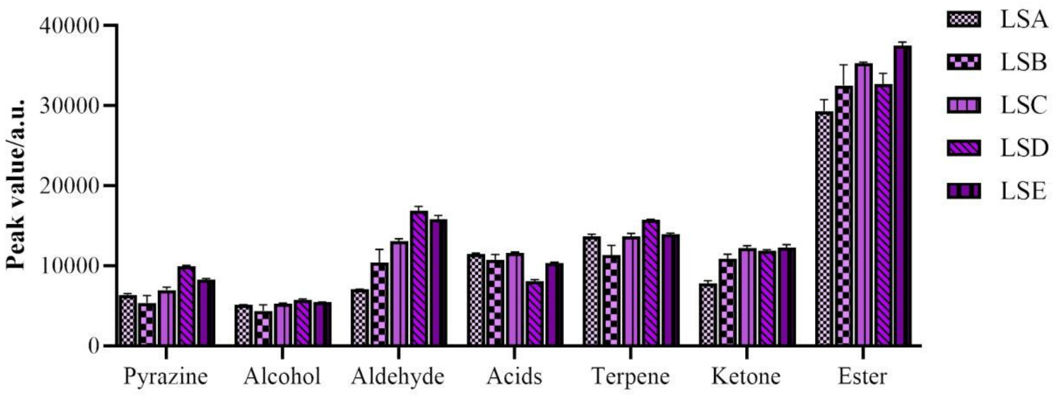

Figure S2. Classification of volatile compound in Sichuan pepper at different harvest periods based on peak volumes
